# Mast cell-derived interleukin-4 mediates activation of dendritic cell during toll-like receptor 2-mediated inflammation

**DOI:** 10.3389/fimmu.2024.1353922

**Published:** 2024-04-30

**Authors:** Joschua Friedel, Sandra Pierre, Anja Kolbinger, Tim J. Schäufele, Blerina Aliraj, Andreas Weigert, Klaus Scholich

**Affiliations:** ^1^ Institute of Clinical Pharmacology, Goethe University, Frankfurt, Germany; ^2^ Institute of Biochemistry I, Faculty of Medicine, Goethe University, Frankfurt, Germany; ^3^ Fraunhofer Institute for Translational Medicine and Pharmacology ITMP, Frankfurt, Germany; ^4^ Fraunhofer Cluster of Excellence for Immune-Mediated Diseases CIMD, Frankfurt, Germany

**Keywords:** mast cells, dendritic cells, high-content immunohistochemistry, inflammatory structure, toll-like receptor 2

## Abstract

**Introduction:**

During an innate inflammation, immune cells form distinct pro- and anti-inflammatory regions around pathogen-containing core-regions. Mast cells are localized in an anti-inflammatory microenvironment during the resolution of an innate inflammation, suggesting antiinflammatory roles of these cells.

**Methods:**

High-content imaging was used to investigated mast cell-dependent changes in the regional distribution of immune cells during an inflammation, induced by the toll-like receptor (TLR)-2 agonist zymosan.

**Results:**

The distance between the zymosan-containing core-region and the anti-inflammatory region, described by M2-like macrophages, increased in mast cell-deficient mice. Absence of mast cells abolished dendritic cell (DC) activation, as determined by CD86-expression and localized the DCs in greater distance to zymosan particles. The CD86- DCs had a higher expression of the pro-inflammatory interleukins (IL)-1β and IL-12/23p40 as compared to activated CD86+ DCs. IL-4 administration restored CD86 expression, cytokine expression profile and localization of the DCs in mast cell-deficient mice. The IL-4 effects were mast cell-specific, since IL-4 reduction by eosinophil depletion did not affect activation of DCs.

**Discussion:**

We found that mast cells induce DC activation selectively at the site of inflammation and thereby determine their localization within the inflammation. Overall, mast cells have antiinflammatory functions in this inflammation model and limit the size of the pro-inflammatory region surrounding the zymosan-containing core region.

## 1 Introduction

Mast cells belong to the first responders to invasive pathogens and can produce a variety of mediators, such as histamine, cytokines, chemokines, growth factors and lipid mediators. While many of these mediators are stored in granules, lipid mediators, e.g. prostaglandin (PG) E_2_, and thromboxane, are generated on demand and are released depending on time and stimulus (1–5). Mast cells are most prominently known for their pro-inflammatory and vasoactive roles, such as key players in allergic and anaphylactic reactions and are mostly found close to regions, which are exposed to the environment, for example mucosal and skin tissue. However, mast cells are also increasingly recognized for their involvement in anti-inflammatory processes. In this regard, it was shown that mast cells can release mediators with anti-inflammatory properties, such as interleukin (IL)-4, IL-10, interferon (IFN)-β or PGE_2_ (4, 6, 7). The release of the different mediators is situation-dependent leading to promotion of either pro- or anti-inflammatory functions in neighboring immune cells. Previously we demonstrated that during late stages of an innate inflammation mast cells are mainly located in an anti-inflammatory environment dominated by M2-like macrophages (4). These mast cells expressed IL-4 and IFN-β, which can induce a switch from the innate to the adaptive immune response thereby effectively reducing innate immune answers (8, 9).

Toll-like receptor 2 (TLR2) is nearly ubiquitously expressed and mediates innate immune responses directed against a wide range of pathogens including Gram-positive bacteria, protozoa and viruses (10–12). Zymosan, a commonly used inducer of TLR2-mediated local inflammation, is arrested at its site of injection due to its a particulate structure. Injection of fluorescence-labeled zymosan can, therefore, be used to define the organization of an inflamed area relative to the pathogen (13–15). The localization of immune cells in regard to zymosan can then be determined using high-content immunohistochemistry technologies, such as Multi-Epitope-ligand-Carthography (MELC). These systems are able to visualize unlimited numbers of antibodies on the same tissue sample and allow statistical analysis of the images leading to a map of the immune cell networks within diseased tissues (14, 16). During a zymosan-induced local inflammation, the major cells of the innate immune system are the major immune cell populations in the inflamed tissue (i.e. neutrophils, eosinophils, macrophages, dendritic cells (DC), mast cells) (14, 15). The structure of a TLR2-mediated inflammation comprises a core-region with the pathogen as well as neutrophils and M1-like macrophages. This core region is surrounded by a pro-inflammatory (PI)-region dominated by M1-like macrophages. The PI-region is then neighbored by an anti-inflammatory (AI)-region characterized by the presence of M2-like macrophages (14, 15). It should be noted that the AI-region may also include pro-resolution processes. The co-existence of pro- and anti-inflammatory microenvironments allows immune cells to fulfill their assigned pro- or anti-inflammatory roles without interfering with cellular functions of immune cells in other microenvironments.

The role of mast cells in the formation of the architecture of an inflammation has not been investigated so far. Here, we used mast cell-deficient Mcpt5-DTA-Cre mice (7) to study the influence of mast cells on the inflammatory structure of a zymosan-induced, local inflammation. We determined the number, phenotype and localization of the immune cells in the inflamed area using high-content imaging and found that in the absence of mast cells, the distance between the pathogen-containing core region and the AI-region increased. An especially strong effect of mast cell-deficiency was a missing alternative activation of DCs and an altered DC localization within the inflammatory structure.

## 2 Material and methods

### 2.1 Mice

Male C57BL/6 mice (6-8 weeks) were provided by Janvier (Le Genest, France). Mast cell protease 5 (Mcpt5)-DTA-Cre mice with C57BL/6N background (6-8 weeks) were previously described (7). Briefly, Cre recombinase expression was controlled by the mast cell-specific Mcpt5 promotor, activating the expression of the catalytically active diphtheria toxin A (DTA) subunit leading to selective depletion of fully differentiated mast cells. As controls for mast cell-deficient Mcpt5-DTA-Cre^+^ mice Mcpt5-DTA-Cre^-^ litter mates were used. Mice were kept according to the International Association for the Study of Pain guidelines. All animal procedures were approved by the local ethics committee (Regierungspräsidium Darmstadt) (Grants FK/1029 and FU/1274). The mice were kept in rooms with controlled climate and light conditions. All mice had free access to food and water.

To induce inflammation, 10 µl zymosan in phosphate-buffered saline (PBS; Merck, Darmstadt, Germany) were injected intraplantar (i.pl.) in a hind paw. Where indicated either 3 or 12 mg/ml were used to allow comparison with a previous report (4). To deplete eosinophils an anti-Siglec F antibody (0.883 mg/kg; clone 238047; R&D Systems, Minneapolis, MN) was injected intraperitoneal (i.p.) 24 hours before the zymosan injection. IL-4c was administered i.p. 24 hours before the zymosan injection and comprised IL-4 (0.166 mg/kg; Peprotech, Hamburg, Germany) and anti-IL4 antibody (0.883mg/kg; Biolegend, San Diego, USA) (14, 17–19). Rat IgG2α (Biolegend, San Diego, USA) was used as control for anti-Siglec F and anti-IL4 antibodies.

### 2.2 Mechanical hypersensitivity

Mechanical paw withdrawal latencies were determined using a plantar aesthesiometer (Dynamic Plantar Aesthesiometer, Ugo Basile) as described previously (20). Here, a 2 mm diameter steel rod is applied to inflamed paws using a force ranging from 0 to 5 g and increasing the force by 0.5 g/s until a withdrawal reaction was observed.

### 2.3 Multi-epitope-ligand-carthography

The MELC technology allows multiple sequential immunohistology for visualization of unlimited numbers of antibodies on one tissue sample (4, 14, 20). Shortly, paw sections (10 µm thick) were placed on silanized cover slips. The tissue was fixed (4% paraformaldehyde in PBS) and permeabilized (0.1% Triton X100 in PBS) each for 15 minutes followed by a blocking step (3% BSA in PBS) for 1 hour. All samples were imaged with an inverted fluorescence microscope (DM IRE2, Leica Microsystems, Wetzlar, GER). Then, a pre-programmed, automated process was used to incubate the samples with bleachable, fluorophore-conjugated antibodies and were afterwards washed with PBS. Next, phase contrast and fluorescence signals of the single antibodies were imaged (Apogee KX4, Apogee Instruments, Logan, USA), followed by a bleaching step to clear fluorescence signals, and recording of a post-bleaching image. This cycle, consisting of incubation, imaging and bleaching, was repeated for the following antibodies. Afterwards all fluorescence images were aligned using the corresponding phase contrast images. Illumination of all images was adjusted using flat-field correction. Finally, post-bleaching images were subtracted from the following fluorescence image to exclude background staining. All antibodies are listed in **Supplementary Table S1**.

### 2.4 Image analysis

The grayscale MELC images were processed using ImageJ v1.52 (NIH, Bethesda, MD, USA) to reduce background fluorescence, and cleared from fluorescence artifacts. For a more precise analysis, the software Cell Profiler v3.1.9 (21) was used to perform an advanced background subtraction, a correction of brightness differences and by a creation of a mask for single-cell segmentation using fluorescent images of propidium iodide (cell nuclei) and CD45 (immune cells). The dataset-dependent segmentation mask was then used for further analysis with the software histoCAT v1.76 (22) together with all corrected fluorescent antibody images. All images, excluding images used to generate the segmentation mask, were z-score-normalized and analyzed by PhenoGraph analysis (23). PhenoGraph clustering was calculated based on their marker expression and classified as different cell types for cell image quantification. The cell number in the clusters was calculated as percent of to the total number of objects. The cell clusters were exported for evaluation using the MATLAB-based program SPADE (v3.0) and visualized as Spanning-trees of density-normalized events using a k-value of 60 (24). To study the neighborhood between cell types, neighborhood analysis was done using the histoCAT software (22) with implemented standard terms. Therefore, pairwise neighborhood of the cell types was calculated for all cells at a radius of 4 pixels and compared to different randomized cell distributions. The included permutation test output is differentiated between a significant neighborhood (P < 0.05), no likelihood or avoidance.

### 2.5 Distance analysis

Distance analyses were performed with ImageJ (Fiji v.2.9.0) (25) as published previously (15). Shortly, the tissue localization of PhenoGraph clusters was visualized by histoCAT and used for the following analysis. Zymosan cluster images were binarized and used to create two-dimensional Euclidian distance map (EDM). Grey values were assigned according to the distance to zymosan. Next, cluster images for specific cell types were binarized and the cells were segmented by watershed algorithm. Determination of cells was done by “analyze particles” function and overlayed on the EDM of the zymosan image. The mean distance between zymosan and the cells was determined by “ROI manager”.

### 2.6 FACS analysis

Cell isolation and preparation was done as described previously (4, 14, 26). Briefly, inflamed paws were cut into <1 mm^3^ pieces and incubated for 45 minutes at 37°C in 500 µl lysis buffer (3 mg/ml Collagenase in RPMI 1640 medium). Lysis was stopped through addition of 5 ml 10% FBS in DMEM. The cells were passed through a cell strainer (70 µm) and incubated in erythrocyte lysis (ACK) buffer for 5 minutes at room temperature. Then the cells were pelleted and blocked in 60 µl of 2% Fc-blocking reagent (Purified Rat Anti-Mouse CD16/CD32 (Mouse BD Fc Bloc); BD Pharmingen, NJ, USA) in PBS for 10 minutes at 4°C. Antibodies (**Supplementary Table S1**) were incubated for 30 minutes at 4°C. Samples were acquired with a flow cytometry system (FACS Canto II; BD Biosciences, Heidelberg, Germany) and analyzed by using FlowJo software v10 (BD Biosciences, Heidelberg, Germany) following the gating scheme shown in **Supplementary Figure S2**. Unstained controls and fluorescence minus one (FMO) controls were used to establish the gating strategy.

### 2.7 Mediator staining in DCs

To determine mediator production in activated dendritic cells, cells inflamed were isolated from paw tissue as described previously (4, 14, 15) and where then treated over night with a 1 µg/ml solution of Brefeldin A (Sigma-Aldrich, St. Louis, USA) in RPMI 1640 medium (Gibco, Carlsbad, USA) to prevent the secretion of cytokines by inhibiting protein transport. After overnight incubation, the residual cells were collected by centrifugation at 1000 g for 5 minutes, and were then divided in two equal cell pellets. Afterwards the cells were incubated with 60 µl of 2% Fc-blocking reagent (Purified Rat Anti-Mouse CD16/CD32 (Mouse BD Fc Block); BD Pharmingen, NJ, USA) in PBS for 10 minutes at 4°C, followed by an extracellular staining with CD45-V500 (BD Pharmingen, Franklin Lakes, USA), CD11c-PerCp (Biolegend, San Diego, USA), CD86-BV421 (Biolegend, San Diego, USA), MHCII-APC (Miltenyi Biotec, Bergisch Gladbach, GER), MHCII-APC-Cy7 (Thermo Fisher Scientific, Waltham, USA), IL-10-FITC (eBioscience, Santa Clara, USA), IL-1β proform-APC-Cy7 (Miltenyi Biotec, Bergisch Gladbach, GER), IL-12(p40)-PE-Cy7 (Biolegend, San Diego, USA), IL-23-PE (BD Pharmingen, Franklin Lakes, USA), IL-6-PE (Biolegend, San Diego, USA), and TNF-α-APC (Miltenyi Biotec, Bergisch Gladbach, GER) for 30 minutes at 4°C. For intracellular staining, residual cells were fixated and permeabilized by using the Cytofix/Cytoperm kit (BD Pharmingen, Franklin Lakes, USA). Afterwards, the cells were stained again with the same antibody master mixes, which are already used for extracellular staining. Samples were acquired with a flow cytometry system (FACS Canto II; BD Biosciences, Heidelberg, Germany) and analyzed by using FlowJo software v10 (BD Biosciences, Heidelberg, Germany). For gating, unstained controls and fluorescence minus one (FMO) controls were used.

### 2.8 Statistical analysis

Statistically significance was calculated using one-way or two-way analysis of variance (ANOVA) by GraphPad Prism v9.0.1. For *post hoc* analysis Tukey correction for multiple comparisons was used. Comparisons between two groups were performed by unpaired two-tailed Student’s t-test. Gaussian distribution was tested by Shapiro-Wilk test. When data were not normal distributed, Mann-Whitney test was used instead of the T- test. F test or Brown-Forsythe test was used to assess the assumption of equal variances.

## 3 Results

### 3.1 Mast cell-deficiency increases the PI-region

**Figure 1:**
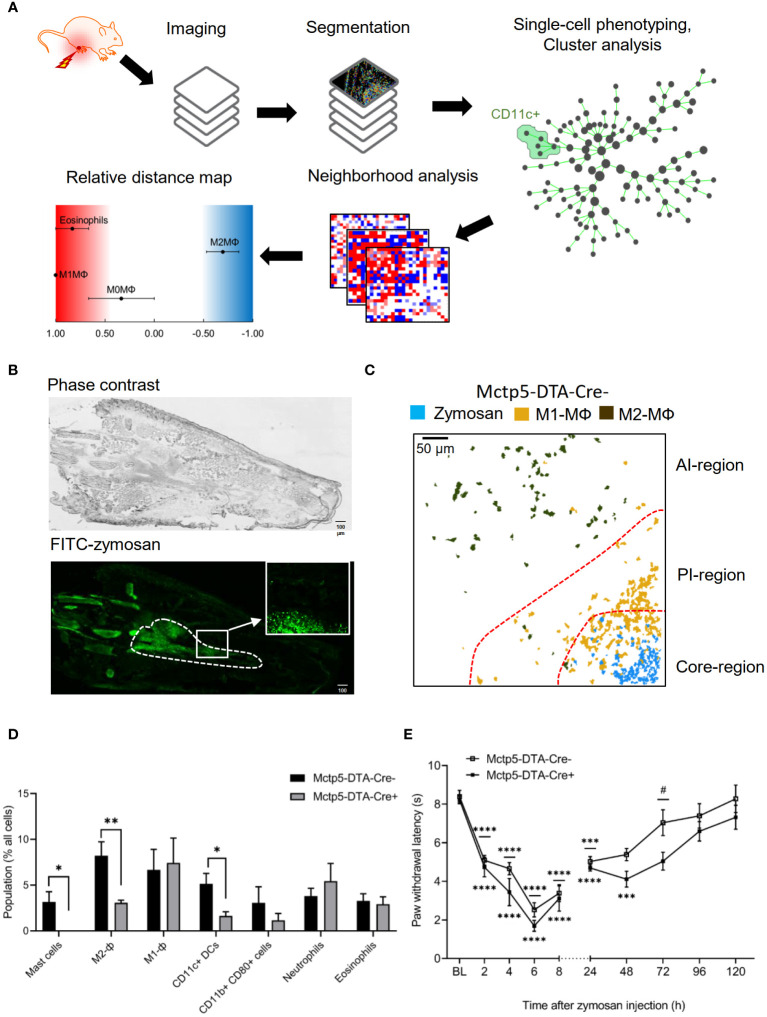
Immune cellular microenvironment of Mctp5-DTA-Cre mice. **(A)** Workflow of the bioinformatic imaging pipeline. **(B)** Representative images showing the distribution of FITC-labeled zymosan (3 mg/ml, 10 µl) in one hind paw. The dotted line indicates the zymosan-containing region (core-region). The inlet shows a typical field of vision used for MELC analysis. Size bar represents 100 µm. **(C)** Composite MELC images showing the position of the core-, proinflammatory (PI)- and anti-inflammatory (AI)-regions in the tissue regarding the localization of zymosan, M1- and M2-like macrophages (MΦ). Red dotted lines depict the area of transition between the neighboring regions. **(D)** Frequency of different immune cell types in the MELC images of Mctp5-DTA-Cre mice after zymosan injection. Data are shown as mean (n = 6 mice) ± SEM (two-tailed Student’s t-test; *p<0.05, **p<0.01). **(E)** Dynamic Plantar test of Mcpt5-DTA Cre- and Cre+ mice during zymosan-induced inflammation. Mechanical paw withdrawal latencies in Mcpt5-DTA Cre- and Cre+ mice (n = 6 mice) at indicated time points after injection of zymosan (10 µl, 12 mg/ml). Data are shown as mean ± S.E.M., One-Way ANOVA, ***p=0.0001, ****p<0.0001, compared to base line, two-way ANOVA with Tukey’s multiple comparison test for determining differences in the resolution phase, # p<0.05.

To characterize the consequences of a mast cell-deficiency on the inflammatory structure, we used mast cell-deficient Mcpt5-DTA-Cre mice. High-content imaging utilizing the MELC technology (14, 16) was performed with 31 antibodies against cytokines, innate immune cells and non-immune cells (**Supplementary Table S1**) to identify the effect of mast cell-deficiency on an acute zymosan-induced innate inflammation. For cell identification segmentation masks were generated depending on nuclear staining together with the immune cell marker CD45 (**Figure 1A**). It should be noted that although all markers were included in the analysis, for better clarity we will only mention the markers, which were used to define specific cell types. After single-cell phenotyping, cell clustering and identification of immune cell types was performed (**Figure 1A**). All visual fields were selected to cover the zymosan-containing region (about 30% of the total image) and the adjacent areas (**Figure 1B**). Visualization of zymosan together with clusters containing M1-like (CD45^+^/Siglec F^–^/F4-80^+^/CD11b^+^/CD86^+^/CD206^–^) and M2-like macrophages (CD45^+^/Siglec F^–^/F4-80^+^/CD11b^+^/CD86^-^/CD206^+^) were used to define the core-, AI- and PI-regions (**Figure 1C**). The three regions form within 24 hours after zymosan injection and persist until pathogen removal is completed (14, 15). In accordance with previous publications (14, 15) we found that 48 hours after zymosan injection the most abundant immune cell clusters in the inflamed paw were neutrophils (CD45^+^/Ly6G^+^/F4-80^–^), macrophages (CD45^+^/Ly6C^–/+^/F4-80^+^/Siglec F^–^/CD11b^+^), DCs (CD45^+^/F4-80^–^/CD11c^+^/MHC II^–/+^) and eosinophils (CD45^+^/Siglec F^+^/F4-80^+^) (**Figure 1D**). Notably, innate lymphoid cells, B-cells, T-cells and NK-cells are not involved in the early stages of zymosan-induced inflammation (<72h after zymosan injection) (14, 15). Since M1-like and M2-like macrophages define the regional structure (14, 15), we divided macrophages into clusters as M1-like macrophages and M2-like macrophages. As expected a complete loss of mast cells was seen in mast cell-deficient Mcpt5-DTA-Cre^+^ mice (**Figure 1D**). In addition, the numbers of M2-like macrophages and DCs were significant reduced in the mast cell-deficient mice (**Figure 1D**). In accordance with the reduced number of M2-like macrophages, we observed in mast cell-deficient mice a mildly increased zymosan-induced hypersensitivity during resolution of inflammation between 48 and 72 hours after zymosan injection (**Figure 1E**).

**Figure 2:**
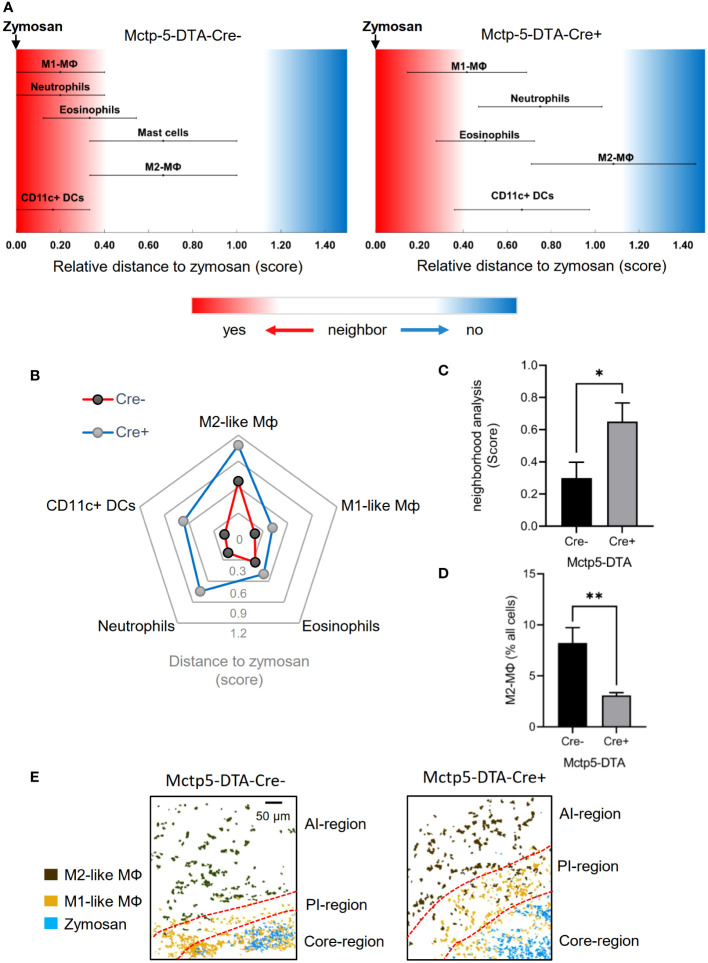
Expansion of Immune cellular microenvironment in Mctp5-DTA-Cre+ mice. **(A)** Relative distance of various immune cell types in Mctp5-DTA-Cre mice based on the likelihood to neighbor zymosan. **(B)** Radar plot of immune cellular microenvironments with M2-like MФ, M1-like MФ, eosinophils, neutrophils and DCs based on the likelihood to neighbor zymosan. **(C)** Neighborhood scores of zymosan with M2-like MФ, M1-like MФ, eosinophils, neutrophils and DCs. Data are shown as mean (n = 6 mice) ± SEM (two-tailed Student’s t-test; *p<0.05). **(D)** Frequency of the M2-like macrophage type in MELC images after zymosan injection. Data are shown as mean (n = 6 mice) ± SEM (unpaired t-test, **p<0.01). **(E)** Composite MELC images showing the position of the core-, proinflammatory (PI)- and anti-inflammatory (AI)-regions in Mcppt5-DTA-Cre mice. The localization of zymosan, M1- and M2-like macrophages (MΦ) are shown. Red dotted lines depict the area of transition between the neighboring regions.

To determine changes in the regional structure of the inflammation caused by absence of mast cells, we analyzed the likelihood of cells neighboring each other as compared to randomized cell distributions (22). For visualization, linear distance maps of the relative distance between zymosan and the identified cell clusters were generated based on scores ranging from 0 (direct neighbors) and 2 (no neighbors) (**Figure 2A**). Identified clusters represented neutrophils, eosinophils, DCs, mast cells as well as M1-like and M2-like macrophages. In mast cell-deficient mice, a general shift of all immune cells towards a reduced likelihood of being neighbored by zymosan was observed, whereby the biggest shifts were seen for M2-like macrophages, neutrophils and DCs (**Figures 2A, B**). Consequently, the average neighborhood score for all 5 immune cell types increased significantly in mast cell-deficient mice (**Figure 2C**). The observed shifts in the neighborhood scores suggest a greater distance to the zymosan particles of at least a part of the immune cells. In the case of M2-like macrophages, which are located in the AI-region at the outer border of the images, such a shift away from the zymosan would position some M2-like macrophages outside of the imaged area. Fittingly, the number of M2-like macrophage in the AI-region was decreased in mast cell-deficient mice (**Figure 2D**). In accordance with a shift of M2-like macrophages to greater distance from the zymosan, visualization of the core-, proinflammatory (PI)- and anti-inflammatory (AI)-regions showed an increase in the size of the PI-region (**Figure 2E**). In regard to neutrophils, FACS analysis based on nuclear propidium iodide staining of neutrophils showed that over 90% of neutrophils are already phagocytosed and over 60% of the remaining neutrophils were apoptotic 24 hours after zymosan injection (**Supplementary Figure S3**). Importantly, in mast cell-deficient mice efferocytosis is decreased due to a diminished phagocytic activity of macrophages (4). Therefore, non-phagocyted neutrophil particles remain for longer time periods in areas where zymosan has already been cleared and, as consequence, reduce the statistical likelihood of zymosan and neutrophils being neighbors.

### 3.2 Mast cell-deficiency alters DC activation and localization at the site of inflammation

**Figure 3:**
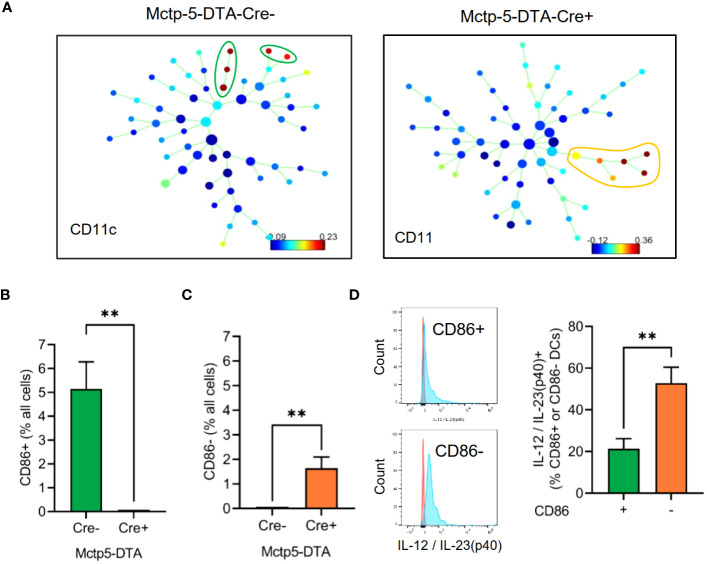
Evaluation of DC phenotypes in Mctp5-DTA-Cre mice. **(A)** Identification of dendritic cell populations using SPADE analysis after zymosan injection. **(B, C)** Frequency of CD86^+^ **(B)** and CD86^-^ **(C)** DCs in MELC images of Mctp5-DTA-Cre mice after zymosan injection. Data are shown as mean (n = 6 mice) ± SEM (two-tailed Student’s t-test, **p<0.01). **(D)** FACS analysis of IL-12/IL-23(p40) produced by CD86^-^ than in CD86^+^ dendritic cells. Data are shown as mean (n = 5 mice) ± SEM (Mann-Whitney test, **p<0.01).

**Figure 4:**
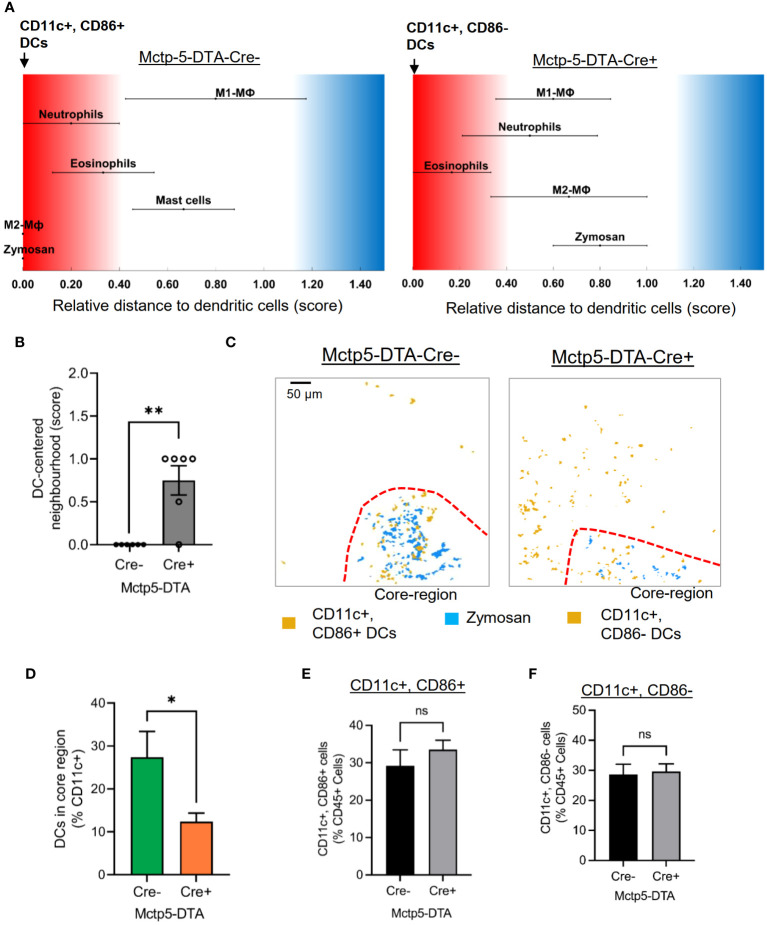
The location of DCs is dependent on its phenotype. **(A)** Relative distance of various immune cell types in Mctp5-DTA-Cre mice based on the likelihood regarding dendritic cells. **(B)** Neighborhood scores for Zymosan and dendritic cells in Mctp5-DTA-Cre mice. Data are shown as mean (n = 6 mice) ± SEM (two-tailed Student’s t-test; **p<0.01). **(C)** Composite MELC images showing the localization of DC clusters in Mctp5-DTA-Cre mice 24 hours after zymosan injection in regard to the core-region. The red dotted lines depict the limit of the core region. **(D)** Frequency of CD11c^+^ dendritic cells in the zymosan based core-region (minimal distance of dendritic cell in 1-5 µm to nearest zymosan particle). Data are shown as mean (n = 6 mice) ± SEM (two-tailed Student’s t-test; *p<0.05). **(E, F)** FACS analysis of CD11c^+^/CD86^+^ **(E)** and CD11c^+^/CD86^-^ **(F)** DCs in inflamed paw tissue of Mctp5-DTA-Cre- and Cre+ mice. Data are shown as mean (n = 6 mice) ± SEM (two-tailed Student’s t-test). ns, non significant.

Regarding DCs, SPADE analyses of the MELC images 24 hours after zymosan injection, generated spanning-trees based on cluster similarity and showed two different DC phenotypes in Cre-negative and -positive mice, which differed in the expression of CD86, a marker for activated or mature DCs (27–30). CD86 was expressed in all DCs in the inflamed area in the paws of Cre-negative control mice (**Figures 3A, B**). In contrast, in mast cell deficient mice only CD86-negative, DCs appeared at the site of inflammation (**Figures 3B, C**). DCs in both genotypes were negative for CCR7, XCR1, CD172a and CD103 (**Supplementary Figure S4**). Previously it was reported that IL-12/IL-23/p40 is expressed by activated DCs and mediates phenotypical changes in T-cell (31–33) and macrophages (34–36). However, FACS analysis of inflamed paws showed that during zymosan-induced inflammation higher percentage of CD86^-^ DCs express IL-12/IL-23/p40 as compared to activated CD86^+^ DCs (**Figure 3D**, **Supplementary Figure S2**). Notably, since IL-12/IL-23p40 can promote M1-like macrophage polarization (34–36), the presence of IL-12/IL-23/p40 expressing DCs at the site of inflammation is in accordance with the extension of the PI-region. In regard to the localization of the two DC populations, comparison of their cellular neighborhoods showed that the CD86^-^ DCs in mast cell-deficient mice have a lower likelihood of being neighbored to zymosan than the activated DCs in Mcpt5-DTA-Cre^-^ control mice (**Figures 4A, B**). This finding was also supported by the finding that activated DCs have a lower frequency of being located in the zymosan-containing core-region (**Figures 4C, D**). Thus, so far the data show that in absence of mast cells the CD86-expression in DCs is abolished and that CD86^-^ DCs are located more distantly to the core-region. Importantly, FACS analysis showed no significant differences in the numbers of CD86^-^ and CD86^+^ DCs isolated out of the entire paw tissue from zymosan-injected Cre-negative and -positive mice (**Figures 4E, F**), suggesting a strictly localized effect of the mast cell-deficiency on DC activation.

**Figure 5:**
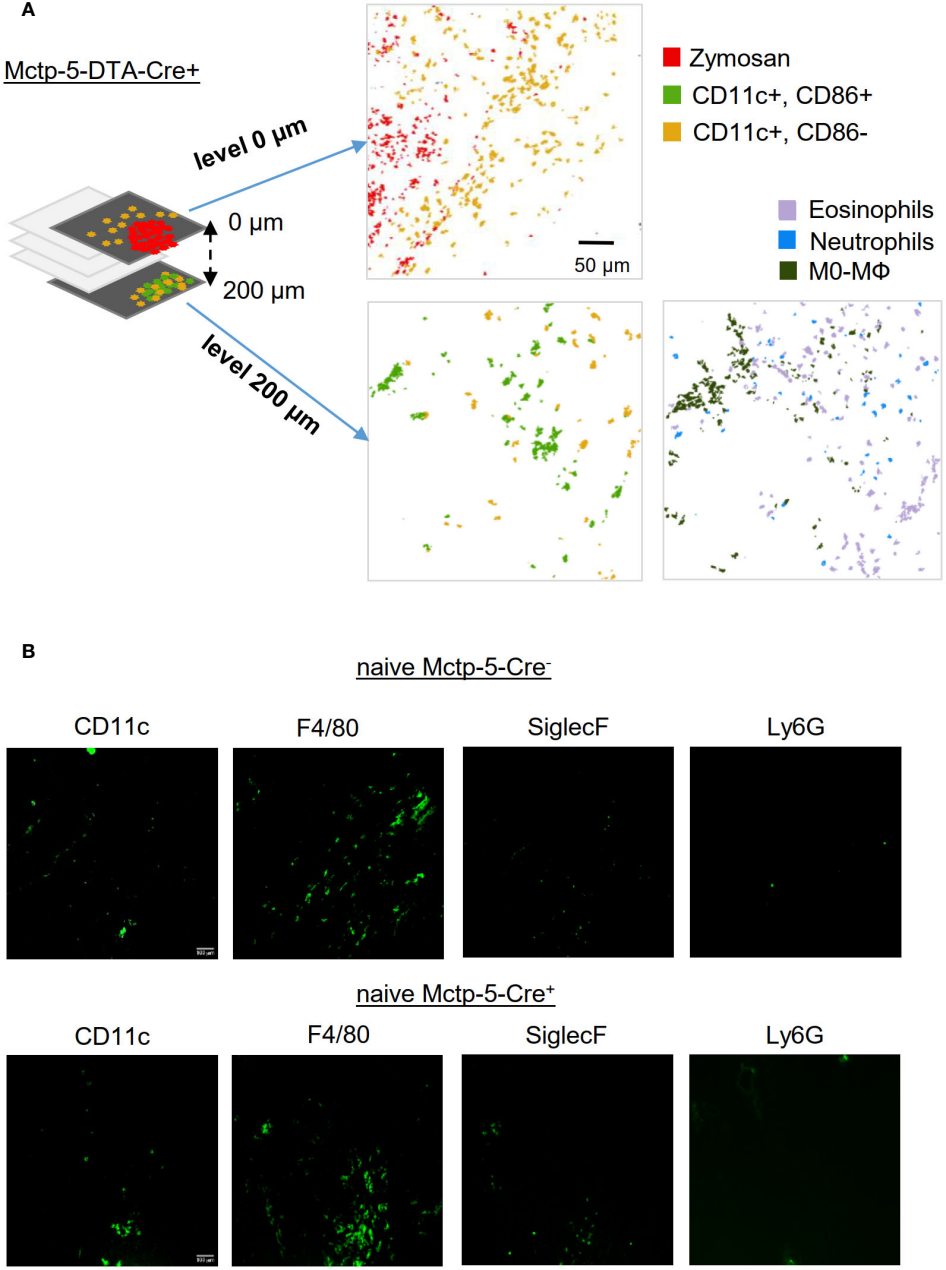
The activation of DCs is just locally altered during the zymosan-induced inflammation. **(A)** Composite MELC images showing the position of dendritic cells in relation to the zymosan particles and most prominent immune cells in serial tissue slices of Mctp5-DTA-Cre+ mice within the zymosan-containing region (level 0 µm) and in a distance from of 200 µm. Size bar represents 50 µm. **(B)** MELC images showing the position of prominent immune cells in healthy paw tissue of Mctp5-DTA-Cre- and Cre+ mice. Size bar represents 100 µm.

Fittingly, MELC analysis of serial tissue slices distant from zymosan particles of mast cell deficient mice revealed that CD86^-^ and CD86^+^ DCs can be observed outside the site of inflammation, demonstrating that mast cell-mediated DC activation is restricted to the inflamed area (**Figure 5A**). Besides DCs also neutrophils, eosinophils and unpolarized macrophages were detected outside of the site of inflammation (**Figure 5A**). In contrast, mainly macrophages and few DCs, but rarely eosinophils or neutrophils were detected in the paw tissue from Cre-negative and -positive mice without zymosan injection (**Figure 5B**). Thus, the strong presence of CD86^-^ and CD86^+^ DCs as well as eosinophils and neutrophils may reflect an increased number of immune cells patrolling the tissue, which surrounds the active inflammation.

### 3.3 IL-4 administration restores activation of DCs

**Figure 6:**
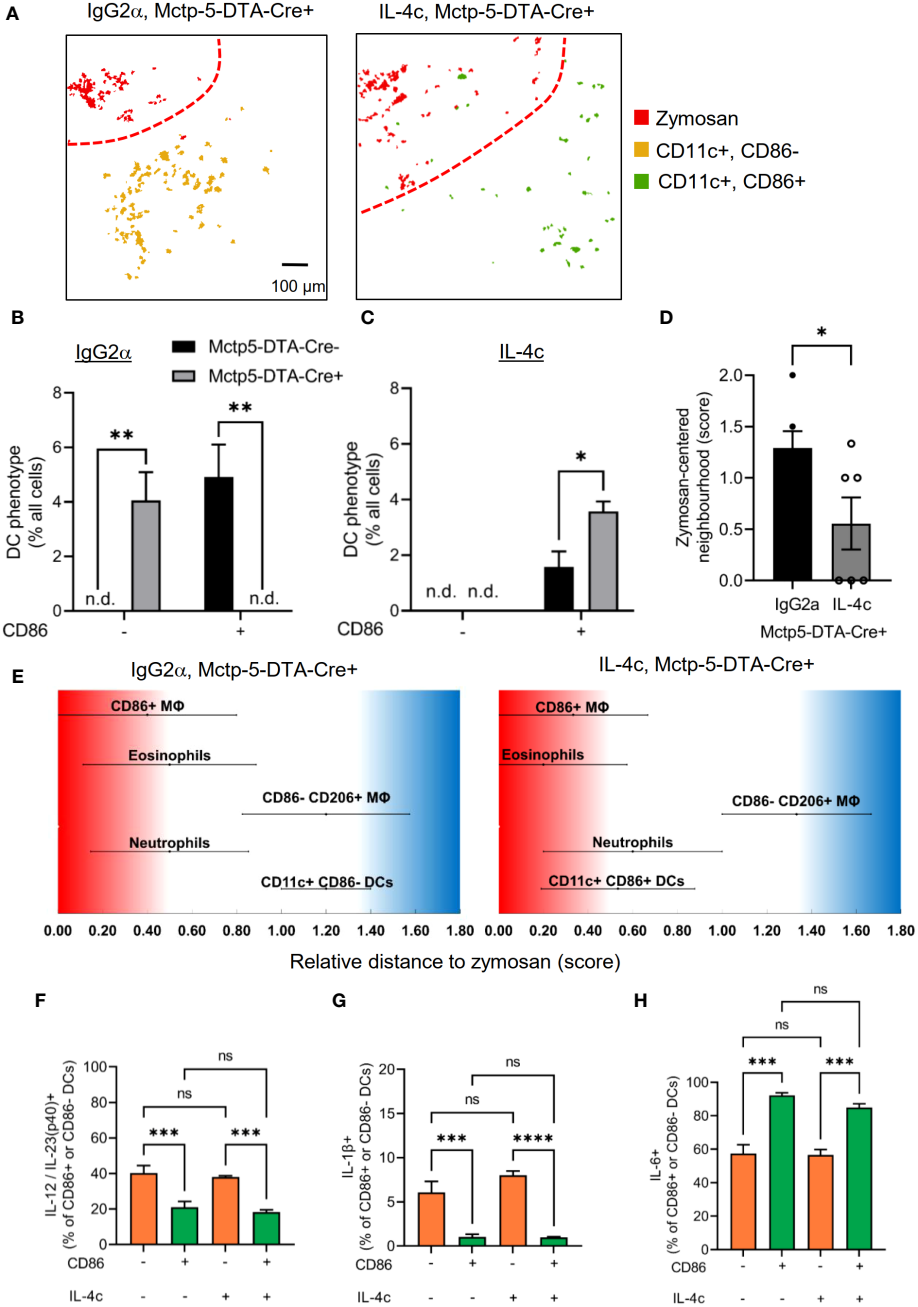
Mast cell derived IL-4 mediates activation and migration of DCs. **(A)** Composite MELC images showing the position of DCs in paw tissue from IL-4c and IgG2α treated Mctp5-DTA-Cre+ mice regarding the zymosan based core-region. Red lines depict the border between core and PI region. Size bar represents 100 µm. **(B, C)** Frequency of CD86^-^ than in CD86^+^ DCs in MELC images of Mctp5-DTA-Cre mice after treatment with IgG2α **(B)** or IL-4c **(C)** together with zymosan. Data are shown as mean (n = 4 mice) ± SEM (two-tailed Student’s t-test, **p<0.01, n.d. = not detected). **(D)** Neighborhood of DCs in IgG2α- and IL-4c pre-stimulated Mctp5-DTA-Cre+ mice based on the likelihood regarding zymosan. Data are shown as mean (n = 6 mice) ± SEM (two-tailed Student’s t-test, *p<0.05). **(E)** Relative distance of various immune cell types in Mctp5-DTA-Cre+ mice based on the likelihood to be neighboring zymosan. (n = 6 mice) ± SEM **(F-H)** FACS analysis of IL-12/IL-23(p40) **(F)**, IL-1β **(G)** and IL-6 **(H)** produced by CD86^-^ than in CD86^+^ DCs in inflamed paws from Mctp5-DTA-Cre^+^ mice. Data are shown as mean (n = 6 mice) ± SEM (two-way ANOVA with Tukey’s multiple comparison test, ***p=0.0001, ****p<0.0001). ns, non significant.

IL-4 is an important inducer of DC activation and maturation, as determined by expression of CD86 (37, 38) and it was previously shown that IL-4 levels are decreased in mast cell-deficient mice during zymosan-induced paw inflammation (4). To determine whether the altered DC maturation in mast cell-deficient mice can be rescued by IL-4 application, we administered a stabilized form of IL-4 (IL-4c) (17–19). Administration of IL-4c was sufficient to change the DC phenotype from CD86-negative DCs to a CD86-positive DC phenotype at the site of inflammation in mast cell-deficient mice (**Figures 6A-C**) while control IgG2α did not influence the DC phenotype in Cre^-^ or Cre^+^ Mcpt5-DTA mice (**Figure 6A**). Also, the neighborhood score, which describes the likelihood of the DCs being neighbors of zymosan, decreased in mast cell-deficient mice after IL-4c administration (**Figures 6D, E**), suggesting a shift of the localization of DCs from the AI region towards the core- and PI-regions. Furthermore, FACS analysis of the inflamed paws showed that IL-4c administration did not alter the expression of IL-23/12p40, IL-1β and IL-6 within the CD86+ and CD86- DC phenotypes (**Figures 6F-H**). Thus, IL-4c administration switches the phenotype of DCs and accordingly also their cytokine profile.

**Figure 7:**
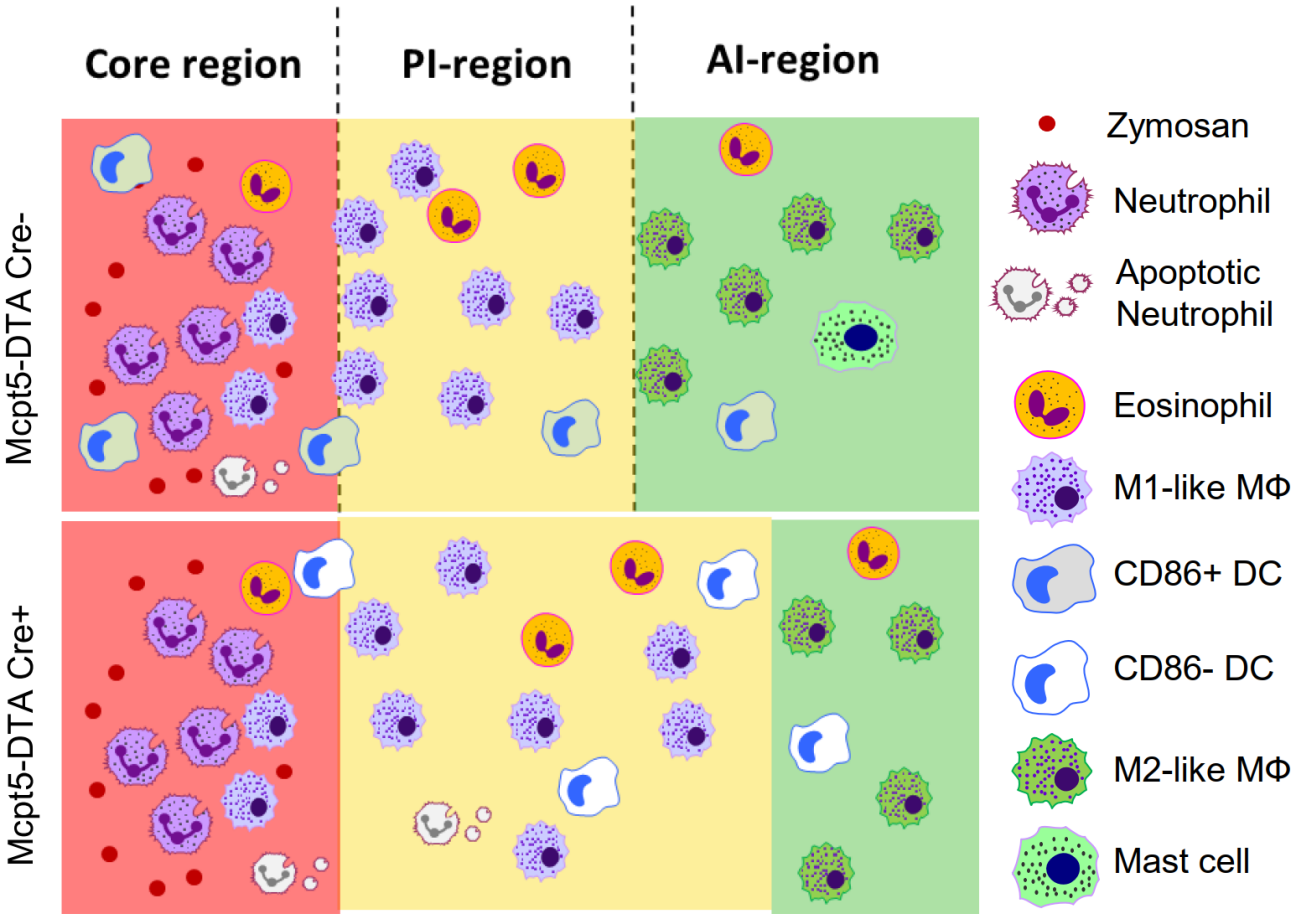
Mast cell-deficiency affects DC activation and localization. Schematic comparison of the inflammatory regions and the localization of the most prominent immune cells between wild type and mast cell- deficiency mice after zymosan injection.

Finally, we studied whether the maturation of DCs during zymosan-induced inflammation can also be mediated by other IL-4-producing cells. In this regard, we previously reported that eosinophils are essential IL-4 producers, which account for around 50% of IL-4 in inflamed paws (14). To test whether the IL-4 effect on DC maturation is specific for mast cells, we reanalyzed previously published MELC data (14) for DC phenotypes in eosinophil-depleted mice with zymosan induced inflammation. Interestingly, we found that eosinophil-depletion did not affect DC activation, demonstrating that IL-4-mediated DC activation at the site of inflammation is indeed a mast cell-specific process (**Supplementary Figures S5A, B**). Taken together, we found that in the absence of mast cells the PI-region widens, DC activation at the site of inflammation is abolished and the localization of DCs within the inflammation depended on their activation state (**Figure 7**).

## 4 Discussion

Inflammation is a process based on a complex immune cell network consisting of inflammation-specific immune cell types and their subpopulations. This cellular network consists of distinct inflammatory regions and, eventually, cellular microenvironments. At later time points (>24 hours after zymosan injection) mast cells are located in the anti-inflammatory AI-region at a considerable distance to the zymosan-containing core-region (4, 14, 15). Mast cells are present in relative small numbers at the site of inflammation during zymosan-induced paw inflammation and their depletion would be expected to have consequences restricted to the cellular microenvironments. However, we found that the likelihood of most cell types neighboring zymosan decreased in absence of mast cells. This resulted in an expansion of the M1-like macrophage-containing PI-region, which separates the core-region from the M2-like macrophage-containing AI-region (**Figure 7C**). In the absence of mast cells decreased anti-inflammatory processes could be expected, since their localization in the AI-region already suggested an anti-inflammatory role and a release of mainly anti-inflammatory and proresolving mediators (4). Which mediators contribute to the expansion of the PI-region in mast cell-deficient mice is unclear, since mast cells produce various mediators with anti-inflammatory or proresolving properties, including IL-4, type 1 interferons or IL-10 (1, 3, 5). The loss of mast cell-derived anti-inflammatory mediators, such as IL-4 within the AI-region, is expected to decrease the presence of anti-inflammatory or proresolving mediators at the transition between AI- and PI-region. s consequence the balance in this transition zone shifts in favor of pro-inflammation and thereby causing the expansion of the PI-region. Thus, this observation underlines the flexible nature of the involved immune processes allowing accommodating answers to changing conditions of the inflammation.

Yet, the extent of the influence of mast cells on the inflammatory structure still surprises and suggests that additional cells are involved in repeating and strengthening their signals. In this regard DCs are a prime target for this function, since their activation is in some inflammatory models mediated by mast cells (39–41). Accordingly, we found that DCs did not develop the CD86^+^ phenotype in mast cell-deficient mice during zymosan-induced inflammation. Notably, cytokine, chemokine and receptor expression differs strongly between mature and immature DCs (34), influencing their activity and migratory properties, whereby CD86 expression in DCs is often used as activation or maturation marker (27–30). Regarding the cytokine profiles of CD86^-^ and CD86^+^ DCs, we found that CD86^-^ DCs expressed IL-12/IL-23p40 and IL-1ß more often, while the CD86^+^ DCs have higher levels of IL-6. IL-12/IL-23p40 can form heterodimers to generate IL-12 or IL-23. Both cytokines promote polarization towards M1-like macrophages (35, 36, 42). It could be argued that IL-1β and IL-12/IL-23p40 released by the CD86^-^ DCs in mast cell-deficient mice, promote M1-like polarization and consequently the expansion of the PI-region. However, IL-6 also fulfills pro-inflammatory roles, but is expressed to a lower extent in CD86^-^ than in CD86^+^ DCs. Since multiple cytokines, which may contribute to the inflammatory process, are differentially expressed in CD86^-^ than in CD86^+^ DCs (34), it is difficult to assess the precise role of one cytokine in the formation of the inflammatory structure. This is further complicated by mediators, such as prostanoids, with pro- and anti-inflammatory effects, depending on the specific inflammatory situation (15). Likewise, the necessity of the formation of immunological synapses for some effects of mediators, such as antigen transfer between mast cells and DCs (39) or DC activation by mast cell-derived IL-4 (39), excludes some mediators from being effective without cell-cell contact, which is *in vivo* difficult to evaluate.

Concerning the absence of CD86^+^ DCs at the site of inflammation in mast cell-deficient mice, it is known that IL-4, which is expressed by mast cells during zymosan-induced inflammation (4), is an essential inducer of DC alternative activation and maturation (37, 38). Fittingly, administration of the stabilized IL-4 restored local DC activation at the inflammation site in absence of mast cells, suggesting that loss of mast cell-derived IL-4 causes the missing DC activation in the inflamed areas. Interestingly, depletion of eosinophils, another important IL-4 source in zymosan-induced inflammation (14), did not alter DC activation. The specificity of mast cell-derived IL-4 in this process might be explained by the necessity of sustained high local IL-4 levels for inducing DC maturation. Notably, mast cells and DCs can form immunological synapses, allowing them to interact over a prolonged time, allowing high local mediator concentrations at the contact points (39). This extended duration of their interaction is necessary, since DCs upregulate IL-4-receptors during the maturation process following their activation (43–45). Eosinophils are not known to form such an extended connection with DCs and, therefore, cannot provide the necessary high IL-4 levels and exposure time for DC activation. Notably, IL-4c has an *in vivo* half-life of ∼24 h and maintains constantly high IL-4 levels for 3–5 days, as compared to an *in vivo* half-life of a few minutes of IL-4, and, therefore, can mimic high local IL-4 releases (18, 19). Since eosinophil depletion did not affect DC activation, eosinophils seem unable to maintain the necessary local IL-4 levels for DC activation. Moreover, besides this direct interaction of mast cells and DCs, mast cell specificity of IL-4 signaling can also be generated by co-stimulation with additional mediators released by mast cells. For example, histamine release is a hallmark of mast cell activation and histamine has been shown previously to be necessary for upregulation of markers for DC activation and differentiation, including CD86, CD14 or HLA-DR (46, 47). The involvement of additional mediators is supported by the finding that IL-4c administration only partly restores DC functions in mast cell deficient mice.

Il-4c administration did not have a major effect on the expression of IL-6, IL12/IL-23(p40) and IL-1β as determined by FACS analysis in DCs isolated from the total paw tissue. In regard to IL-6 it has been shown that IL-4 can suppress the upregulation of IL-6 in response to type I interferons (48, 49) whereby IL-6 production in unstimulated DCs was either not affected (48) or slightly increased (49). Similarly, stimulation with ligands for TLR3 and TLR9 showed no induction of IL-6 in GM-CSF/IL-4 differentiated bone marrow-derived DCs, while they induced IL-6 expression in DCs differentiated with Flt3 ligand (50). Also, another publication demonstrated that IL-4 does not affect unstimulated or LPS-stimulated IL-6 synthesis in bone marrow-derived DCs (51). Likewise, no effect of IL-4 on IL-12/IL-23(p40) expression was seen in unstimulated DCs, while a small inhibitory effect was seen after LPS stimulation (51). Taken together, IL-4 seems not to have major effects on the investigated cytokines in DCs in absence of other stimuli. Since the majority of DCs are outside of the inflamed area (defined as combined area of core, PI and A region), it can be assumed that they are not stimulated by the zymosan or type I interferons and that only a limited effect of IL-4c administration on the cytokine content exists.

The localization of CD86^-^ than in CD86^+^ DCs differs between Mcpt5-DTA Cre^-^ and Cre^+^ mice in so far that CD86^-^ DCs were positioned more distant to the zymosan-covered core-region. This is in accordance with the differential expression of chemokine receptors (e.g. CXCR4, CCR1, CCR2, CCR5, CCR6, fMLPR, C5aR) in mature and immature DC populations, which predisposes them to respond to the various mediators with a distinct positioning in the inflamed tissue (34). Thus, either a specific receptor or the combination of several receptors are decisive for directing migration towards the core region. Fittingly, IL-4c-induced DC-activation restored at least partly the localization of the DCs in regard to the neighborhood of zymosan. Notably, IL-4 has been shown to mediate DC migration indirectly through alterations in the expression of chemokine, cytokine and other receptors (52–54),. Thus, mast cell-derived IL-4 is probably first changing the activation status of DCs and subsequently the localization within the inflamed tissue. This IL-4 dependent change of DC localization in regard to zymosan is based on an increased number of DCs in the direct vicinity of zymosan, leading to an overall increased likelihood of DCs and zymosan to be neighbors. Since this is due to an increased invasion of the core region by DCs and the field of visions chosen for the analysis are covering only a small portion of the core region, most of the DCs in the core region are not included in the field of visions. This is reflected by the reduced CD86^+^ DC numbers in the MELC analysis.

In conclusion, our present data show that mast cells can influence the inflammatory structure in a more far-reaching manner than their specific localization and relative low number at the site of inflammation might suggest. The most striking effect of the mast cell-deficiency was the disappearance of the alternatively activated CD86^+^ DC phenotype and affecting the inflammatory structure even in areas where mast cells are not located. Thus, mast cell-induced DC activation may relay mast cell signals, thereby increasing the impact of mast cells on the inflammatory structure, whereby additional cellular neighbors affected by from mast cells, such as M2-like macrophages, may also serve in amplifying their signals. Taken together, characterization of the immune cell networks underlying the inflammatory structure allows to identify the impact of cells on the cellular network beyond its direct cellular neighborhood allowing a deeper understanding of the underlying pathomechanisms. These insights can then be used to identify the mechanism of action of drugs (15), predict therapy resistance in patients (55) and predict outcome prognosis (56, 57).

## Data availability statement

The raw data supporting the conclusions of this article will be made available by the authors, without undue reservation.

## Ethics statement

The animal study was approved by local ethics committee Regierungspräsidium Darmstadt. The study was conducted in accordance with the local legislation and institutional requirements.

## Author contributions

JF: Data curation, Formal analysis, Investigation, Methodology, Writing – review & editing. SP: Resources, Supervision, Writing – review & editing. AK: Formal analysis, Investigation, Methodology, Writing – review & editing. TS: Investigation, Methodology, Supervision, Writing – review & editing. BA: Investigation, Methodology, Writing – review & editing. AW: Funding acquisition, Methodology, Resources, Supervision, Writing – review & editing. KS: Conceptualization, Funding acquisition, Project administration, Supervision, Writing – original draft.
