# Supplementary material for: Mast cell-derived interleukin-4 mediates activation of dendritic cell during toll-like receptor 2-mediated inflammation
Source: Front Immunol. 2024 Apr 30;15:1353922. doi: 10.3389/fimmu.2024.1353922 (PMC11091258; doi:10.3389/fimmu.2024.1353922)
Supplement: Supplementary file 1 [file DataSheet_1.docx]

Supplementary Material

Mast cell-derived interleukin-4 mediates activation of dendritic cell during toll-like receptor 2-mediated inflammation

Joschua Friedel, Sandra Pierre, Anja Kolbinger, Tim J. Schäufele, Blerina Aliraj, Andreas Weigert, Klaus Scholich*

*** Correspondence:** Corresponding Author: scohlich@em.uni-frankfurt.de

**Supplementary Table S1.** Antibodies used for MELC and FACS analysis


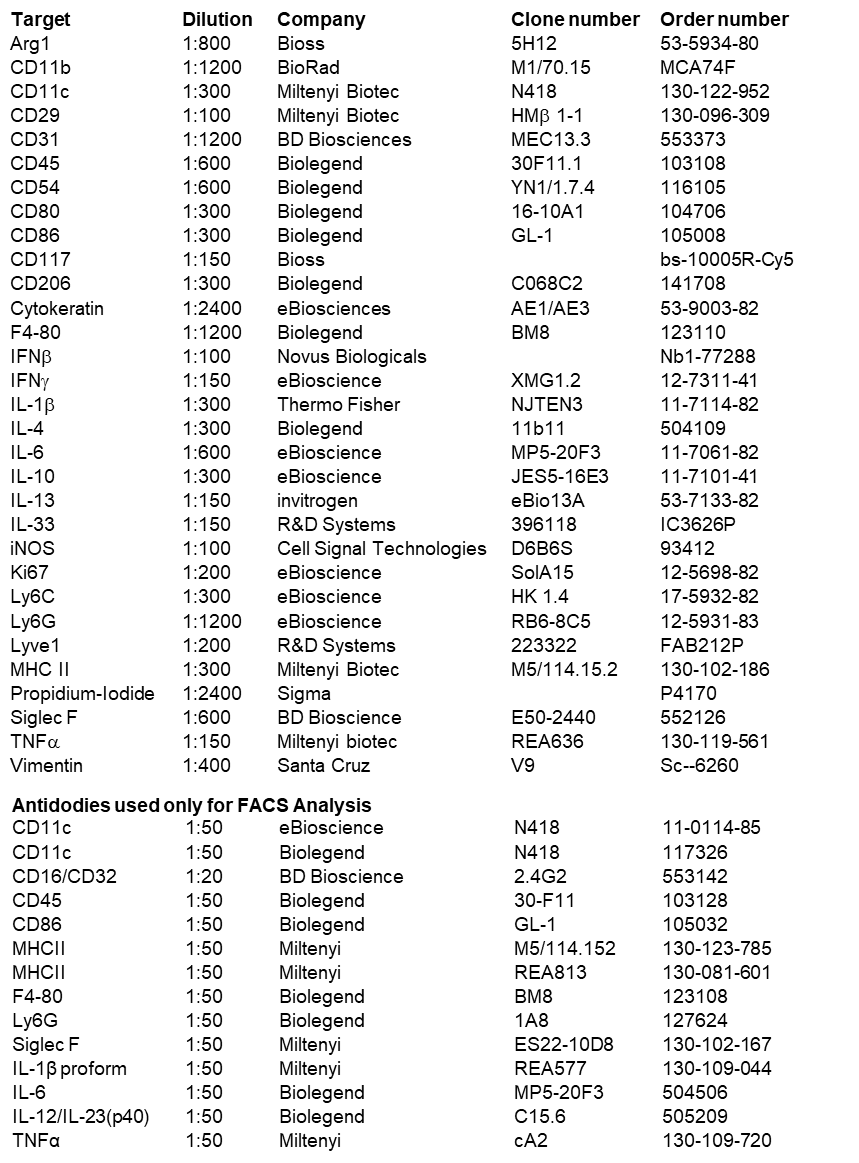


**Supplementary Figure S2: FACS Gating strategy for immune cells and effector assays of dendritic cells after injection of zymosan.**

Cells were gated based on their side scatter area (SSC-A) and forward scatter area (FSC-A). Then, CD45^+^ cells were identified. Ly6G^+^/F4/80^-^ cells were defined as free neutrophils and Ly6G^+^/F4/80^+^ cells as efferocytosis of neutrophils by macrophages. Red arrows depict the gating strategy for determining efferocytosis.

**
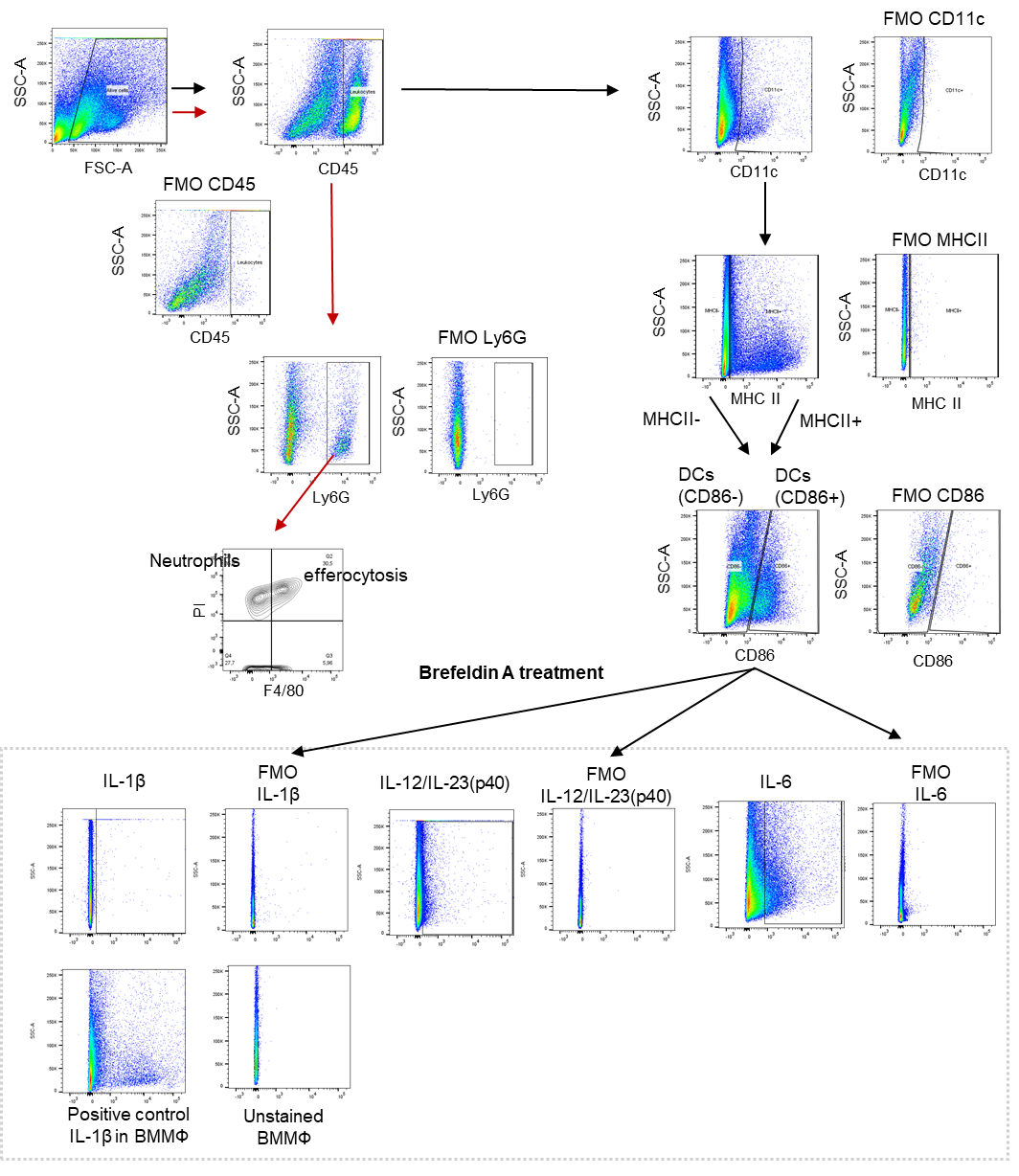
**Using an independent antibody panel (black arrows) we defined within DCs (CD11c^+^) cells, activated dendritic cells as CD11c^+^/CD86^+^ and non-activated DCs were defined as CD11c^+^/CD86^-^. IL-1β, IL-6, and IL-12/IL-23(p40) were determined in CD86^-^ and C86^+^ DCs. For IL-1β a positive control and its unstained control are shown using bone marrow-derived macrophages. Images are generated using Mcpt5-DTA-Cre^-^ mice.

**Supplementary Figure S3: 24 hour after zymosan injection the majority of neutrophils is apoptotic or phagocytosed.**

**(A,B)** FACS analysis of F4 80^-^/Ly6G^+^ cells for the number of non-apoptotic (panel A) and apoptotic (panel B) neutrophils 24 after zymosan injection (3 mg/ml) into the paws of Mcpt5-DTA-Cre^-^ mice. Data are shown as mean ± SEM (n = 4). **(C)** FACS analysis of Ly6G+ cells for the number of neutrophils phagocyted by macrophages 24 after zymosan injection (3 mg/ml) into the paws of Mcpt5-DTA-Cre^-^ mice. Data are shown as mean ± SEM (n = 4).

**
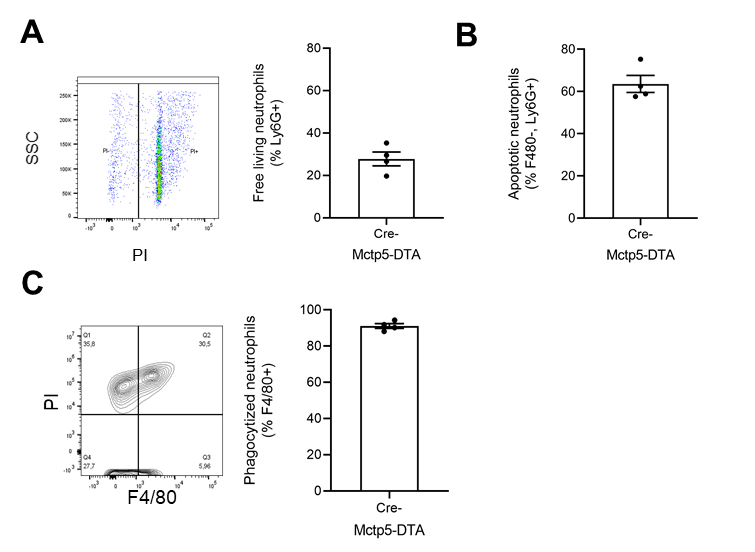
**

**Supplementary Figure S4: DCs in the inflamed area were negative for CCR7, XCR1, CD172a and CD103.**

Representative MELC analysis of DCs for expression of CCR7, XCR1, CD172a and CD103 24 hours after zymosan injection (3 mg/ml, 10 µl) in paws of Mcpt5-DTA Cre^-^ and Cre^+^ mice. CCR7 and XCR1 were expressed in mast cells but not in DCs.

**
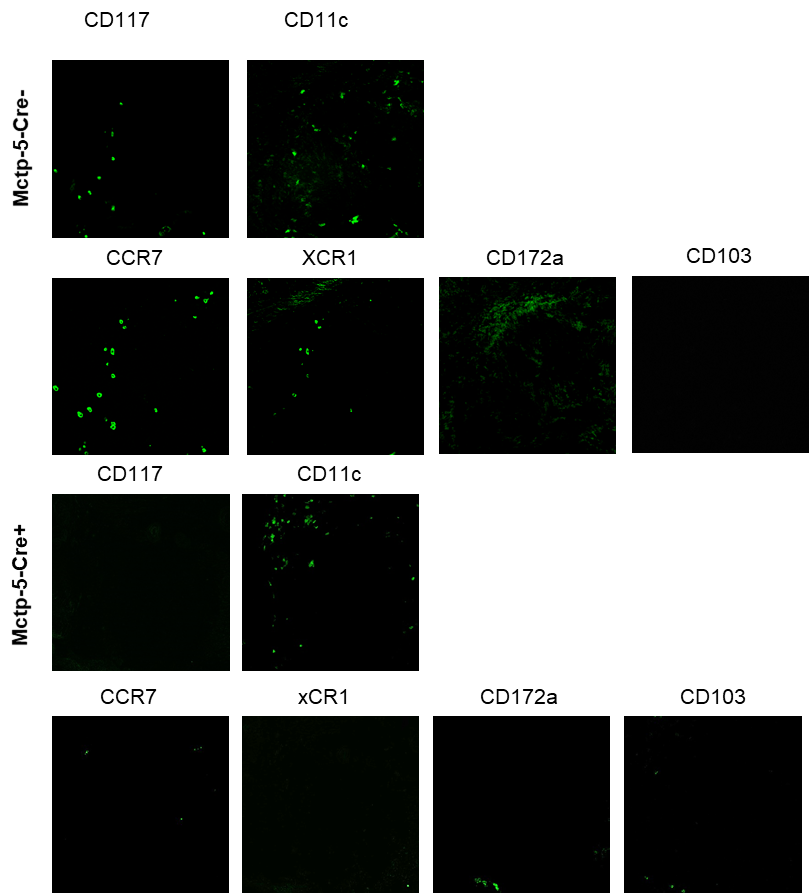
**

**Supplementary Figure S5: Eosinophils are not responsible for a successful activation of DCs during the zymosan-induced inflammation.**

**(A,B)** MELC analysis of DC phenotypes without eosinophil depletion using an anti-IgG2α (panel A) or anti-SiglecF antibody (panel B) at 24h or 48h after zymosan injection. Data are shown as mean (n = 6 mice) ± SEM (two-tailed Student’s t-test).

**
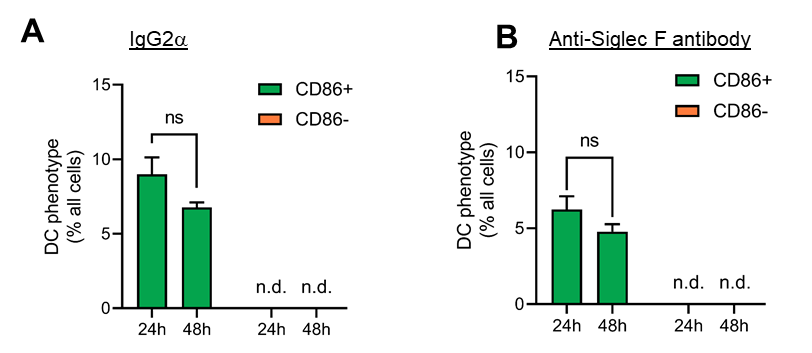
**
